# Olympic Combat Sports as Weight-Management Interventions in Non-Athlete Children and Adolescents: A Systematic Review

**DOI:** 10.3390/sports14090378

**Published:** 2026-09-01

**Authors:** Guanwen Zhou, Hrvoje Karninčić, Ivan Segedi, Ivan Todorov, Damir Pekas

**Affiliations:** 1 Faculty of Sport and Physical Education, University of Nis, 18000 Niš, Serbia; zhouguanwen26@gmail.com; 2 Faculty of Kinesiology, University of Split, 21000 Split, Croatia; hrvojek@kifst.hr; 3 Faculty of Kinesiology, University of Zagreb, 10000 Zagreb, Croatia; ivan.segedi@kif.hr; 4 European Judo Union, 1146 Budapest, Hungary; ivan.todorov@eju.net

**Keywords:** combat sports, taekwondo, judo, children, adolescents, body composition

## Abstract

Background: The official combat sports in the Olympic Games include fencing, taekwondo, wrestling, judo, boxing, and karate. Most current research indicates that participation in Olympic combat sports significantly improves the physical fitness of children and adolescents, but specific studies on its weight loss effects in this population remain relatively limited. This systematic review evaluated controlled Olympic combat-sport interventions for weight management and body composition in non-athlete children and adolescents. Methods: We searched five databases and platforms: PubMed, ProQuest, EBSCOhost, Web of Science (Core Collection), and Embase, up to 3 November 2025. Eleven reports met the eligibility criteria. Results: Four reports involved overweight or obese youth (three taekwondo and one judo intervention), and seven involved normal-weight participants (six judo and one taekwondo intervention). The studies differed substantially in populations, comparators, outcome definitions, and reporting. Reported within-group changes in body mass, BMI, and adiposity-related outcomes cannot alone establish comparative intervention effects. Among overweight or obese youth, the available taekwondo reports described favourable changes in selected adiposity-related outcomes, whereas evidence for judo and findings among normal-weight participants were inconsistent. In normal-weight children, changes in absolute body mass require interpretation alongside growth, maturation, and body-composition measures. Conclusions: The available evidence is limited, heterogeneous, and requires design-appropriate risk-of-bias reassessment. It does not support conclusions about fencing, boxing, wrestling, or karate. Future controlled trials should report prespecified, age- and sex-adjusted outcomes, complete between-group analyses, and adverse events.

## 1. Introduction

Adolescent obesity has become a major public health problem worldwide. Obesity increases cardiovascular disease and other health risks, affecting the physical and mental health of adolescents. Exercise intervention can be an effective means of reducing the body fat percentage of overweight and obese adolescents [1]. As one of the origins of modern combat sports, wrestling was practiced by many outstanding philosophers in ancient Greece to improve their physique. At the same time, wrestling is also considered an activity with important educational significance for children’s growth [2]. The official combat sports events of the modern Summer Olympic Games include fencing, taekwondo, wrestling, judo, boxing, and karate. Except for karate, which only appeared once, at the 2020 Tokyo Olympics, the other five are permanent Olympic events.

Currently, multiple systematic reviews and meta-analyses in the academic community have shown that Olympic combat sports have an important impact on people’s physical and mental health. Valdés-Badilla et al. pointed out that older adults can effectively improve their physical function, physiological and mental health by participating in comprehensive training in Olympic combat sports [3]. In addition, these sports also have varying degrees of benefits for the quality of life of healthy men and women, patients with Parkinson’s disease and patients with breast cancer [4]. The study by Munoz-Vasquez et al. further showed that such training can improve the cardiopulmonary function of healthy non-athlete populations of different ages and significantly increase their maximum oxygen uptake (VO_2_max) [5].

Some scholars have conducted a systematic analysis of relevant research on the participation of children and adolescents in Olympic combat sports. Hernandez-Martinez et al.’s research showed that Olympic combat sports can improve the standing long jump performance of non-athlete students but have no significant effect on cardiopulmonary function [6]. At the psychological level, Lee et al.’s research pointed out that Olympic combat sports interventions help improve the mental health of disabled children and adolescents [7].

Some scholars have also conducted research and analysis on specific individual events in Olympic combat sports. Gutierrez-Garcia et al. believe that judo training can not only increase the bone mineral content of participants’ arms, but also improve physical fitness indicators such as flexibility, muscle endurance and agility, and help avoid subcutaneous fat accumulation [8]. Piepiora conducted a special analysis of karate and believed that karate has certain positive effects on health, but research on its long-term health effects is still insufficient [9]. In addition, other studies have further confirmed that judo-specific training for school-aged children has a positive effect on their health [10], especially showing significant benefits in terms of muscle strength, endurance, speed, coordination and flexibility [11]. Taekwondo training is an effective form of exercise that can prevent or improve obesity [12]. It is also an intervention that can effectively maintain the health of the elderly and improve their physical fitness [13].

While most studies indicate that Olympic combat sports have positive effects on physiological indicators and can significantly improve the physical fitness of children and adolescents, specific research on their weight-loss effects in this population remains relatively limited. Therefore, this article aims to systematically review published research on Olympic combat sports interventions in children and adolescents, analyze the specific impacts of these sports on weight management in children and adolescents, and attempt to compare the differences in effects between obese and normal-weight children and adolescents, thereby providing a reference for the development of education and public health policies.

## 2. Materials and Methods

This research protocol has been registered on PROSPERO (Registration number: CRD420251271709).

### 2.1. Database Search Strategy

This search utilized five general-purpose databases and platforms: PubMed, ProQuest, EBSCOhost, Web of Science (Core Collection), and Embase. The Proquest platform searched two databases, APA PsycArticles, APA Psyclnfo, while the EBSCOhost platform searched CINAHL Ultimate, MEDLINE Complete, ERIC and SPORTDiscus with Full Text. During the search process, we employed the following main relevant search terms: [Topic] (“boxing” OR “fencing” OR “judo” OR “karate” OR “taekwondo” OR “wrestling” OR “Olympic combat sports”) AND [Topic] (“free fat mass” OR “fat mass” OR “body mass index” OR “lean body mass” OR “body fat percentage” OR “visceral fat” OR “body weight” OR “overweight” OR “obesity”) AND [Topic] (“children” OR “child” OR “schoolchildren” OR “young” OR “youth” OR “adolescents” OR “young people” OR “teenagers”). Due to the differences in databases, there are also certain variations in the search formulas. The specific search formulas can be found in the Supplementary Materials. Additionally, due to the different times of database establishment, there are also certain differences in the search periods. The start of the search period is the time of database establishment, and the end of the search period is 3 November 2025. The specific search periods can also be found in the Supplementary Materials. Eligible reports were restricted to publications available in English. This restriction was applied so that the review team could conduct full-text screening, data extraction, and methodological appraisal consistently and reliably in English. The restriction was specified as an eligibility criterion and applied during study selection.

### 2.2. Eligibility Criteria

The inclusion criteria for this systematic review were research articles published in peer-reviewed journals. Only articles in English were included. Given the widespread popularity of Olympic combat sports globally, and the existence of related research articles in other languages, including only English articles in our study presents certain limitations. Review articles, conference proceedings, commentaries, and literature in other languages were not included. Furthermore, article inclusion followed the PICOS principle (population, intervention, control group, outcome measure, and study design) (Table 1). This review examined the effects of Olympic combat-sport interventions on weight management and body-composition outcomes in non-athlete children and adolescents with normal weight, overweight, or obesity. Studies involving participants with medical conditions that could independently affect body mass, body composition, growth, or the safe participation in exercise were excluded. This criterion was intended to reduce clinical confounding and to ensure that the reported outcomes could be interpreted primarily in relation to the intervention rather than to an underlying condition. No minimum intervention duration was applied as an eligibility criterion. Nevertheless, all studies that met the inclusion criteria evaluated interventions lasting at least 10 weeks.

### 2.3. Study Selection

Two authors independently reviewed the titles and abstracts of the retrieved studies to determine their eligibility for inclusion. If a determination was still undecided, a third author was involved to resolve the issue and make the final decision. Additionally, we consulted Google Scholar and Research Gate to identify other studies that met the inclusion criteria, ensuring no omissions.

### 2.4. Data Summary

We obtained and analyzed the following data from the selected studies: researcher names and publication year; age and gender data of the samples; number of participants in the experimental and control groups; training content in the experimental and control groups; total training duration, frequency, and duration of each training session; and relevant indicators such as weight and BMI in the studies.

## 3. Results

**Figure 1:**
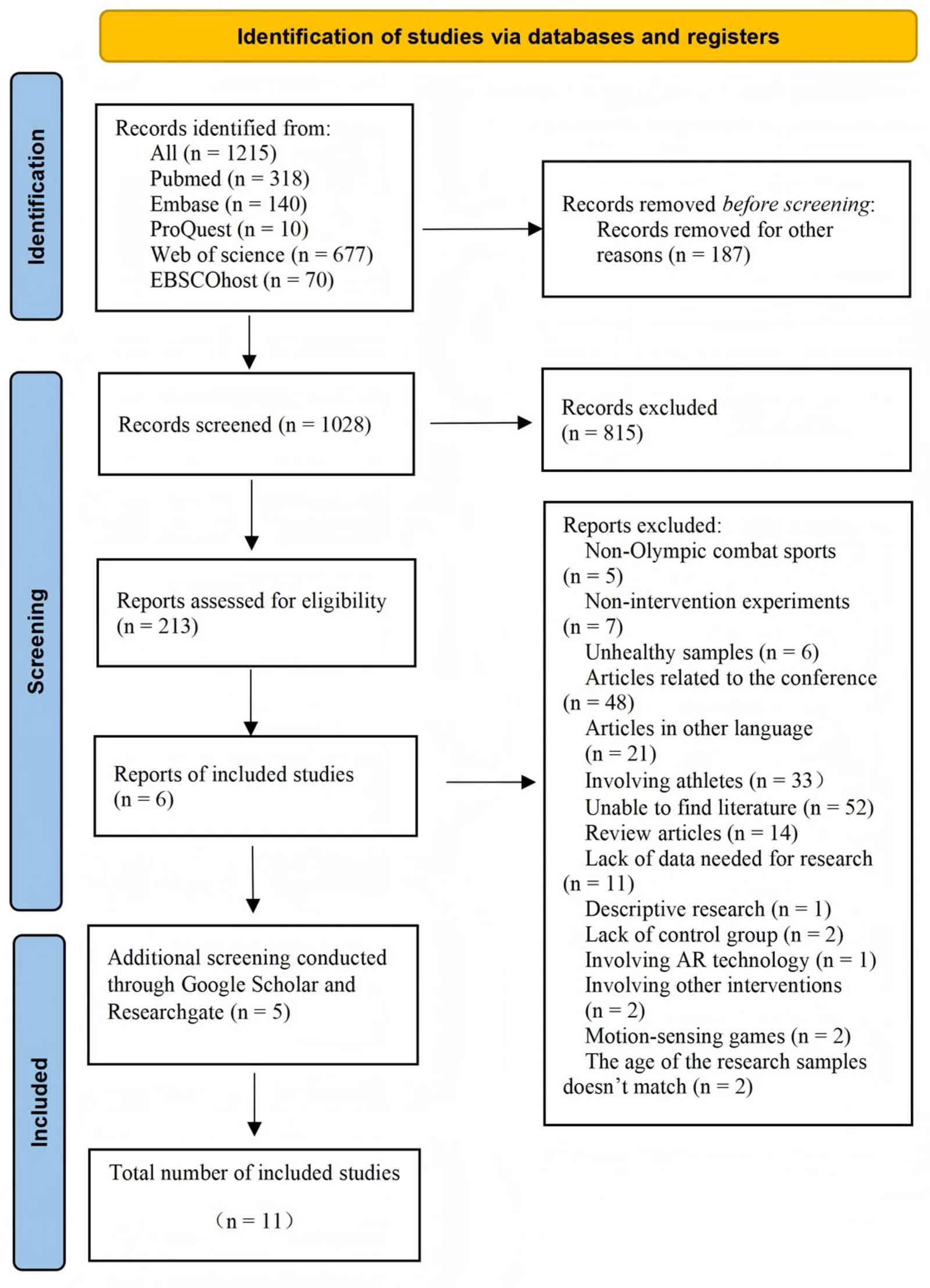
PRISMA 2020 flow diagram.

This systematic review was completed in accordance with the guidelines for system review reports, PRISMA 2020 statement [14]. Our detailed search strategy is shown in Figure 1. A total of 1215 relevant studies were retrieved through electronic database searches. After removing duplicates (187 articles), 1028 articles remained. Subsequently, based on the title, keywords, and abstract, 815 studies irrelevant to the topic were excluded. Finally, 213 studies were included for detailed analysis. Each study was reviewed and screened based on its research subjects, methods, and results. Based on the inclusion criteria and screening conditions, we excluded studies on non-Olympic combat sports, studies irrelevant to the experiment, studies involving unhealthy samples, studies with athletes as research subjects, conference-related studies, and studies in other languages. Subsequently, we further excluded 11 articles lacking necessary data, 1 article containing only descriptive statistics, 2 articles lacking a control group, 1 article involving augmented reality (AR) technology, and several other categories of articles. Following the database searches, we conducted supplementary manual searches to identify potentially eligible records that may not have been retrieved through the electronic search strategy. Google Scholar was used as an additional search source, using the same core concepts as the database searches. ResearchGate was not used as a bibliographic database; rather, it was used to contact authors or request access to full-text articles when potentially eligible reports could not be obtained through institutional or other available sources. As shown in Figure 1, among the 11 reports finally included, 6 were identified through the main database search, while the remaining 5 were determined through supplementary manual searches (Google Scholar) and contact with the authors (ResearchGate). Each study was presented using the following indicators: references (authors and publication year), age and gender, sample size (experimental group and control group), duration, frequency, time, training content, and indicators.

### 3.1. Risk of Bias Within Studies

**Figure 2:**
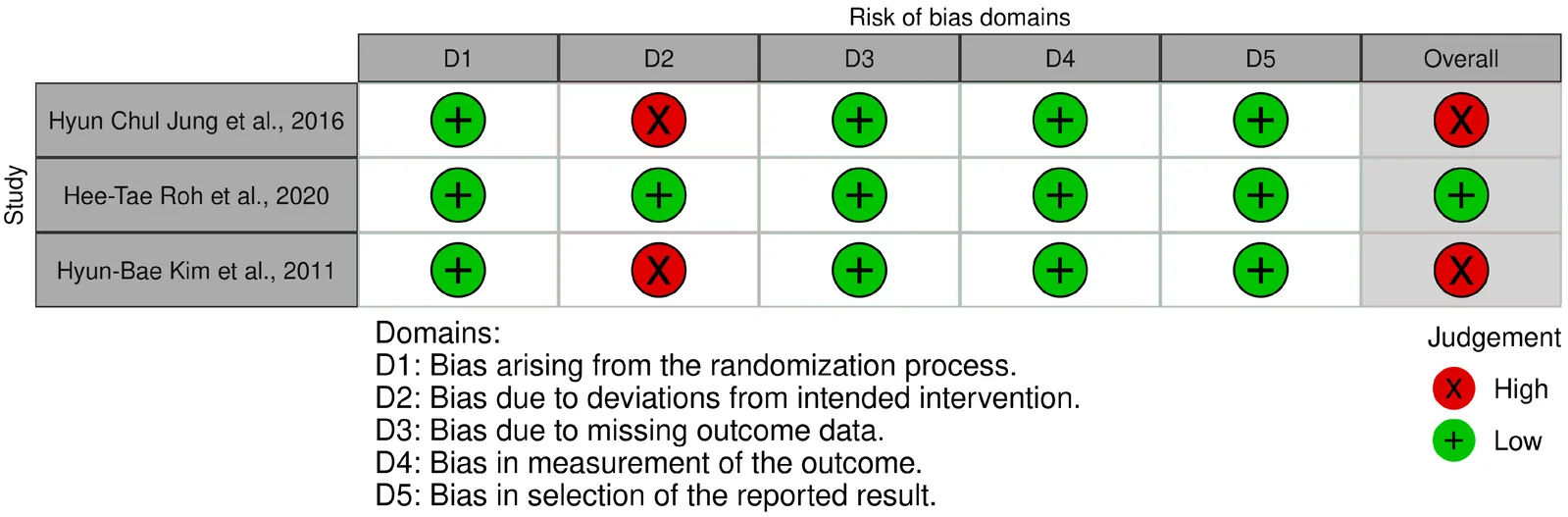
ROB2 bias diagram [15,16,17].

**Figure 3:**
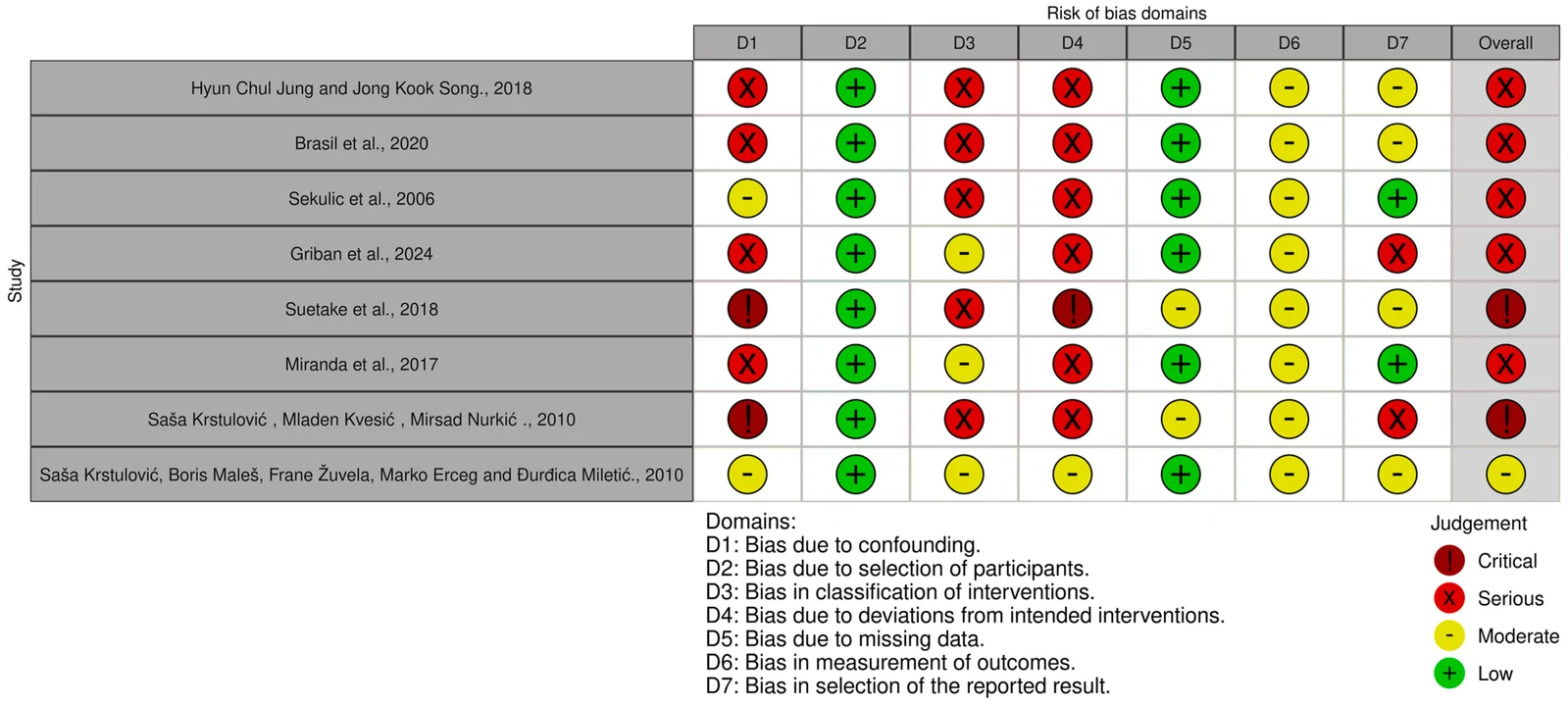
ROBINS-I Bias diagram [18,19,20,21,22,23,24,25].

Two reviewers independently assessed risk of bias for each included study and resolved disagreements through discussion; where consensus could not be reached, a third reviewer adjudicated. Risk of bias was assessed separately according to study design. RoB 2 was used for the three randomized controlled trials, whereas ROBINS-I was used for the eight non-randomized studies of interventions. Figure 2 shows the assessment results of the included RCTs. Among the three included RCTs, only one study [15] (Roh et al., 2020) was rated as “low risk” in all five assessment dimensions, indicating a relatively low risk of bias. In contrast, the other two studies [16,17] (Jung et al., 2016 and Kim et al., 2011) were overall rated as “high risk”, mainly due to methodological flaws in the “deviation from expected intervention measures” (D2) dimension. Figure 3 shows the assessment results of the included non-randomized intervention studies. Among the eight included non-randomized intervention studies, the overall risk of bias was more severe, and no study was rated as “low risk”. Five of the eight studies were rated as “high risk”, and two studies [18,19] (Suetake et al., 2018 and Krstulović, et al., 2010) were rated as “critical risk” due to fatal flaws in the control of confounding factors, and only one study [20] (Krstulović, et al., 2010) was rated as “moderate risk”. The core factors contributing to the increase in the risk of bias in non-randomized studies mainly concentrated in the “confounding” (D1) domain, while the overall performance in the “selection of study participants” (D2) domain was good.

### 3.2. Studies Characteristics

Due to the limited number of studies meeting the inclusion criteria and the lack of research on wrestling, fencing, boxing, and karate, our included literature only covers taekwondo and judo. This is a limitation of our study. As shown in Table 2 and Table 3, the 11 research articles we included, 3 were from Republic of Korea, 1 from The United States of America, 1 from Ukraine, 3 from Brazil, and 3 from Croatia. The total sample size for this study was 633 individuals. Among them, 98 were obese or overweight children and adolescents, including 23 girls; and 535 were children and adolescents of normal weight, including 165 girls. In Table 3, there are four studies on overweight and obesity that meet the requirements of this paper. Most of the participants were secondary school students aged 12 to 15. Only one study included primary school students aged 8 to 13. This study also had the largest sample size, with 20 participants in the OB group and 15 participants in the normal weight control group [22]. The study with the smallest sample size had a total of 20 participants in the experimental and control groups [15,21]. In terms of intervention methods, taekwondo was the most commonly used method, used in three studies [15,16,21]; judo was used in one study [22]. The study using taekwondo as an intervention method had the longest intervention period, 16 weeks [15,16,21]. The intervention period for the study using judo as an intervention method was 12 weeks [22]. Three reports evaluated 16-week taekwondo interventions compared with no exercise [15,16,21], whereas one report evaluated a 12-week judo intervention [22]. The three taekwondo reports described reductions from baseline to follow-up in body mass and BMI within the intervention groups; the report by Jung and Song also described reductions in fat tissue, while body-fat percentage and lean tissue showed no statistically significant pre–post change according to the reported notation [21]. The judo report described no statistically significant pre–post change in body mass or BMI, but reported decreases in fat mass, trunk fat, and body-fat percentage, alongside an increase in lean mass [22].

Table 3 shows studies involving children and adolescents of normal weight. A total of seven studies met the requirements of this paper. Most of the studies involved children in primary school. The largest study had 68 participants in the experimental group and 55 in the control group [20]; the second largest study had 65 participants in the experimental group and 40 in the control group [25]. The smallest study had 31 participants in the experimental and control groups [17]. Six reports evaluated nine-month judo programmes [18,19,20,23,24,25], and one report evaluated a 12-week taekwondo programme [17]. Comparator conditions varied substantially and included mini-sport programmes, regular physical education and other sports, music/art/computer classes, no training, and no additional exercise. According to the reported table notation, body mass and BMI generally showed no statistically significant pre–post change, whereas selected studies reported changes in adiposity-related outcomes, including body-fat percentage, fat body mass, and the sum of two skinfolds [17,19,20,23,25].

**Table 2:** Characteristics of included studies on obese and overweight children and adolescents.

Study		Sample			Duration	Intervention	CON	Main Results
Author (Year)	Country	Age/Years	Gender	Population				
Jung et al., 2016 [16]	Republic of Korea	13–15	B	CG (*n* = 12) CON (*n* = 11) All: 23	16 wk	taekwondo	No exercise	↓*—BW pre–post CS 84.6–80.7, pre–post CON 86.4–86.9 ↓*—BMI pre–post CS 28.9–27.0, pre–post CON 30.6–30.2;
Jung & Song, 2018 [21]	The United States of America	12–15	B	CG (*n* = 11) CON (*n* = 9) All: 20	16 wk	taekwondo	No exercise	↓*—BW pre–post CS 86.8–82.6, pre–post CON 84.6–85.7; ↓*—BMI pre–post CS 29.1–27.2, pre–post CON 29.7–29.5; ↔—% body fat pre–post CS 33.4–30.1, pre–post CON 35.7–34.3; ↓*—Fat tissue pre–post CS 28.4–24.8, pre–post CON 29.4–28.7; ↓*—Lean tissue pre–post CS 54.6–54.3, pre–post CON 51.4–53.3;
Roh et al., 2020 [15]	Republic of Korea	12.55 ± 0.51	B & G	CG (b/g) (*n* = 10) (7/3) CON (b/g) (*n* = 10) (7/3)	16 wk	taekwondo	No exercise	↓*—BW pre–post CS 58.34–55.99, pre–post CON 54.99–55.34; ↓*—BMI pre–post CS 24.91–23.59, pre–post CON 23.74–23.82;
Brasil et al., 2020 [22]	Brazil	8–13	B & G	OB (b/g) (*n* = 20) (10/10) EU (b/g) (*n* = 15) (8/7) All: 35	12 wk	judo	No exercise	↔—BW; pre–post: 54.6–55.4; ↔—BMI; pre–post: 24.6–24.5; ↓*—Fat mass pre–post: 22.8–22.3; ↓*—Trunk Fat pre–post: 10.1–9.8; ↑*—Lean mass pre–post: 29.6–31.9; ↓*—% Fat pre–post: 42.4 39.7;

Legend: B—boys; G—girls; CG—combat group; CON—control group; wk—week; OB—overweight or obese; EU—eutrophic; ↑*—significant increased; ↓*—significant decreased; ↔—no significant difference; BW—body weight.

**Table 3:** Characteristics of included studies on normal weight children and adolescents.

Study		Sample			Duration	Intervention	Comparator	Main Results
Author (Year)	Country	Age/Years	Gender	Population				
Sekulic et al., 2006 [23]	Croatia	7.1 ± 0.3	B	CG (*n* = 41) CON (*n* = 57)	9 m	judo	Mini-soccer, Mini-basketball, Mini-handball	↔—BW pre–post CS 27.7–29.9; pre–post CON 26.9–29.3; ↑*—SUM2SF pre–post CS 16.6–16.4; pre–post CON 17.1–19.1
Kim et al., 2011 [17]	Republic of Korea	15.7 ± 0.6	G	CG (*n* = 21) CON (*n* = 10)	12 wk	taekwondo	No other exercise	↔—BW; ↔—BMI; ↓*—BF% pre–post CS 31.2–29.0; pre–post CON 31.2–30.8; ↓*—FBM pre–post CS 16.8–15.7; pre–post CON 17.7–17.8; ↔—LBM pre–post CS 34.8–35.8; pre–post CON 36.0–36.6
Yahupov V et al., 2024 [24]	Ukraine	16–17	B	CG (*n* = 27) CON (*n* = 27)	9 m	judo	Various sports	↔—BW pre–post CS 70.9–71.4; pre–post CON 71.2–72.1; ↔—BMI pre–post CS 23.1–22.9; pre–post CON 22.9–22.9;
Suetake et al., 2018 [18]	Brazil	9.05 (±2.02)	B & G	judo (boys/girls) (*n* = 21) (9/12) CON (boys/girls) (*n* = 24) (18/6)	9 m	judo	Music, art, and computer classes	↔—BW pre–post CS 44.1–43.0; pre–post CON 36.5–38.8. ↔—BMI pre–post CS 20.40–19.40; pre–post CON 19.4–19.5
Miranda et al., 2017 [25]	Brazil	5–15	B & G	judo (boys/girls) (*n* = 65) (38/27) CON (boys/girls) (*n* = 40) (30/10)	9 m	judo	No training	↔—BW ΔMean CS 2.63; CON 2.16; ↔—BF% ΔMean CS −1.06; CON −0.02; ↑*—BFkg ΔMean CS 0.15; CON 1.36;
Krstulović et al., 2010 [19]	Croatia	7	G	CG (*n* = 30) CON (*n* = 49)	9 m	judo	Mini-volleyball, Mini-basketball, Mini-handball	↔—BW pre–post CS 26.3–28.7; pre–post CON 26.7–29.0; ↑*—SUM2SF pre–post CS 24.0–23.2, pre–post CON 25.7–27.3;
Krstulović et al., 2010 [20]	Croatia	7	B	judo (*n* = 68) CON (*n* = 55)	9 m	judo	Regular PE classes, soccer, track and field	↔—BW; ↔—SUM2S; CG ↑*

Legend: B—boys; G—girls; CG—combat group; CON—control group; wk—week; m—months; ↑*—significant increased; ↓*—significant decreased; ↔—no significant difference; BW—body weight; BF—body fat; SUM2SF—sum of 2 skinfolds; LBM—lean body mass; FBM—fat body mass.

## 4. Discussion

This systematic review examined the effects of Olympic combat-sport interventions on weight management and body-composition outcomes in children and adolescents with normal weight, overweight, or obesity. The review included 11 study reports and considered body mass, BMI, and adiposity-related outcomes, including body-fat percentage, fat mass, lean mass, and skinfold measures. This broader outcome framework is important because body mass and BMI alone may not adequately reflect intervention-related changes in growing children and adolescents.

Several included studies reported increases or no significant changes in body mass despite favourable changes in adiposity-related outcomes. Such findings should be interpreted in the context of normal growth and maturation, as well as possible changes in lean tissue. Consequently, reductions in body-fat percentage, fat mass, trunk fat, or skinfold thickness may provide more clinically meaningful information than changes in body mass alone, particularly among normal-weight participants. The interpretation of these outcomes nevertheless requires caution because the included studies differed in participant characteristics, intervention type and duration, comparator conditions, outcome measures, and methodological quality. Regarding the effects of taekwondo on overweight and obese adolescents, all three articles using taekwondo as an intervention reported that the weight and BMI of the experimental groups decreased significantly, and the differences were statistically significant [15,16,21]. Three articles indicated a significant decrease in weight and BMI in the experimental group, insufficient for concluding that taekwondo caused greater weight reduction than the comparator. This is similar to the results of a systematic review on the effects of combat sports on body composition in overweight and obese children and adolescents, which indicated that adolescents can effectively reduce their BMI by participating in combat sports for at least 12 weeks [26]. Due to differences in exercise intensity and methods, the intervention effects of taekwondo and judo also differ. A study using judo as an intervention indicated that the pre- and post-test comparisons of the obese group showed a certain upward trend in maximum body mass index values, but the difference was not statistically significant, and BMI did not change significantly [22]. This judo study included children aged 8–13 years, representing the primary school student population. As the article states, both judo and taekwondo have a significant effect on the body fat content of the subjects, effectively reducing indicators such as fat mass and body fat percentage [21,22]. This also confirms the conclusion of a previous review article that judo can prevent the increase in subcutaneous fat in participants [8].

Regarding the impact of interventions on children of normal weight, three studies using judo as an intervention reported pre- and post-measurement weight data of the study samples, showing a significant increase in weight with statistical significance [19,20,23]. Two of these studies reported significant differences in the SUM2SF index between the experimental and control groups, but no significant differences were found in the pre- and post-measurement comparisons within the experimental group itself [20,23]. In addition, another study reported a certain downward trend in the SUM2SF index between pre- and post-measurements, but no significant difference was found [19]. The samples in these three studies were all primary school students around 7 years old. In another study that also focused on primary school students, a certain downward trend in weight and BMI was reported, but it was not statistically significant [18]. The observed increases in body mass may partly reflect normal growth and maturation; however, this interpretation cannot be confirmed from the available data. For adolescents, the intervention of judo did not have a significant impact on weight and BMI [24]. In studies covering both children and adolescents, a certain increase in weight was reported, but it was not statistically significant, and there were no significant differences in the effects on related indicators such as fat [25]. Therefore, it may be concluded that the intervention of judo will not have a significant effect on weight loss in children and adolescents with normal weight, and the changes in related indicators such as fat are also small. In addition, studies that use taekwondo to intervene in adolescents reported that the body fat ratio and maximum fat mass of the experimental group samples decreased significantly and were statistically significant [17].

Overall, the available taekwondo studies reported favourable changes in body-composition outcomes among children and adolescents with overweight or obesity and among normal-weight participants, relative to their respective comparison groups. These findings included reductions in body mass and BMI in studies involving overweight or obese participants and reductions in selected adiposity-related measures, such as body-fat percentage and fat mass, across the included populations. However, the evidence base was small and heterogeneous, and the reported outcome data and comparator conditions varied across studies. Therefore, these findings should be interpreted as suggestive rather than definitive evidence of a comparative benefit of taekwondo for body composition.

In contrast, the available judo evidence did not demonstrate consistent effects on body mass or BMI. In the single included study of children with overweight or obesity, judo participation was associated with favourable changes in adiposity-related outcomes, including reductions in fat mass, trunk fat, and body-fat percentage, despite no statistically significant change in body mass or BMI. This pattern is clinically relevant because adiposity-related measures may provide a more informative indication of body-composition change than body mass alone in growing children. Nevertheless, the conclusion regarding judo is based on only one study in overweight or obese participants and must be interpreted cautiously. Additional well-designed, adequately powered controlled trials are needed to determine whether judo produces reproducible improvements in body composition in this population. This is consistent with the conclusion of the previous review that organized judo courses have a positive impact on the health of school-aged children, and these beneficial changes are reflected in body fat, lean body mass and other aspects [10].

Based on our selection criteria, this study focused only on children and adolescents who had not previously participated in related sports, thus excluding studies involving adolescent athletes. Overall, the number of studies meeting the inclusion criteria was limited, and there was a lack of intervention-controlled studies specifically targeting wrestling, fencing, boxing, and karate training. This reflects the current state of research in this field. Moreover, this review was limited to English-language publications and may therefore be subject to language bias. Relevant studies published in other languages may have been missed. Although the restriction enabled consistent full-text screening, data extraction, and critical appraisal by the review team, the findings may not fully represent all available international evidence. Existing research on fencing, wrestling, boxing, and related sports largely focuses on athlete, while studies targeting children and adolescents as sports intervention subjects are relatively few. Furthermore, due to the different research focuses of the included studies, the relevant outcome measures also differed. Only a few studies reported in detail multiple fat-related indicators; most studies only described changes in weight and BMI before and after testing. This is a major limitation of this study. Weight loss research should not only focus on changes in weight and BMI; future research needs to further explore changes in other body composition indicators (such as bone, water, and muscle) and combine these indicators with differences in fat-related indicators to comprehensively evaluate the intervention effects of Olympic combat sports on weight management in children and adolescents from a holistic perspective. Furthermore, it is crucial to emphasize that weight reduction in competitive combat sports is rarely based on a reduction in subcutaneous adipose tissue; instead, it frequently relies on short-term and often health-hazardous dehydration procedures aimed at qualifying for a lower weight category, making such practices entirely inapplicable and unacceptable for the general population. Conversely, combat sports are energetically highly demanding, and their training systems can serve as a valuable tool for gradual and health-justified body reduction in fat mass.

A major source of heterogeneity across the included studies was the type of comparator condition. Control groups ranged from no exercise or no training to regular physical education, recreational sports, various sport programmes, and non-exercise activities such as music, art, or computer classes. These conditions are not methodologically equivalent and may materially influence the observed magnitude and direction of differences between intervention and comparison groups.

Comparisons with no exercise or no training may capture the effect of participating in additional structured physical activity, rather than an effect unique to taekwondo or judo. Conversely, regular physical education, recreational sports, and other sport programmes constitute active or partially active comparators; any smaller difference between groups may therefore reflect a similar physical-activity stimulus in both groups rather than the absence of benefit from the combat-sport intervention. Non-exercise comparator activities may provide attention and social contact but do not necessarily match the exercise dose, energy expenditure, or movement demands of the intervention. Consequently, the findings should not be interpreted as a single pooled estimate of the effect of Olympic combat sports.

Future controlled trials should define comparator conditions more precisely, report the physical-activity exposure received by both groups, and distinguish clearly between inactive, usual-care, and active exercise comparators. This would allow a more valid assessment of whether any observed changes in adiposity-related outcomes are attributable to the specific combat sport, to structured physical activity more generally, or to differences in total exercise exposure.

## 5. Conclusions

The available evidence indicates that structured taekwondo and judo interventions may support favourable changes in body composition among children and adolescents with overweight or obesity, particularly through reductions in adiposity-related outcomes such as body-fat percentage, fat mass, and trunk fat. These outcomes are more informative than absolute body mass alone, because body mass in growing children may change with normal growth, maturation, and gains in lean tissue. Among normal-weight children and adolescents, the evidence does not support weight loss as a primary objective; rather, combat-sport participation should be interpreted in relation to body composition, physical development, and healthy growth. However, the evidence base remains small, heterogeneous, and limited by differences in study design, comparator conditions, outcome reporting, and risk of bias. Importantly, the included controlled intervention studies evaluated only taekwondo and judo; no eligible evidence was identified for fencing, boxing, wrestling, or karate. Well-designed, sport-specific controlled trials are therefore needed to determine whether other Olympic combat sports can safely and effectively improve adiposity-related and broader health outcomes in children and adolescents.

## Figures and Tables

**Table 1:** Inclusion and exclusion criteria for the literature.

Category	Inclusion Criteria	Exclusion Criteria
Population (P)	Children and adolescents who are obese, overweight, and normal weight, and without other major diseases that could affect the implementation of interventions or outcome assessment.	Non-students or students with neurodevelopmental disorders (such as autism). Athletes.
Intervention (I)	Interventions with Olympic combat sports (judo, fencing, karate, boxing, taekwondo, wrestling) for 10 weeks or more.	Non-Olympic combat sports intervention. Other interventions (such as nutrition, lifestyle guidance, etc.).
Comparator (C)	Interventions with a control group.	Studies without a control group.
Outcome (O)	Including relevant indicators such as weight and body fat percentage. Include at least one of the following indicators: weight or BMI. The frequency, intensity, type, and duration of the exercise are clearly specified.	Lack of data.
Study design (S)	Experimental design studies (randomized controlled trials and non-randomized controlled trials), including pre- and post-test evaluation data.	Cross-sectional, retrospective, and prospective observational cohort study.

## Data Availability

The data that support the findings of this study are available from the corresponding author, upon reasonable request.

## Supplementary Materials

The following supporting information can be downloaded at: https://www.mdpi.com/article/10.3390/sports14090378/s1, Table S1: Search strategy for PubMed (318); Table S2: Search strategy for Embase (140); Table S3: Search strategy for ProQuest (10) Database: APA PsycArticles; APA Psyclnfo; Table S4: Search strategy for Web of Science (677); Table S5: Search strategy for EBSCOhost (70) Database: CINAHL Ultimate; MEDLINE Complete; ERIC; SPORTDiscus with Full Text.
