# Supplementary material for: Olympic Combat Sports as Weight-Management Interventions in Non-Athlete Children and Adolescents: A Systematic Review
Source: Sports (Basel). 2026 Sep 1;14(9):378. doi: 10.3390/sports14090378 (PMC13611690; doi:10.3390/sports14090378)
Supplement: Supplementary file 1 [file sports-14-00378-s001.zip › sports-4463737-supplementary.pdf]

sports

MDPI

Supplementary Materials

Supplementary S1 Search strategy

Table S1. Search strategy for PubMed (318).

Database/platform.	Date(year-month-day)
PubMed	Start to End:<br>1975-01-01 to 2025-11-03

(("boxing"[All Fields] OR "fencing"[All Fields] OR "judo"[All Fields] OR "karate"[All Fields] OR "taekwondo"[All Fields] OR "wrestling"[All Fields] OR "Olympic combat sports"[All Fields]) AND ("free fat mass"[All Fields] OR "fat mass"[All Fields] OR "body mass index"[All Fields] OR "lean body mass"[All Fields] OR "body fat percentage"[All Fields] OR "visceral fat"[All Fields] OR "body weight"[All Fields] OR "overweight"[All Fields] OR "obesity"[All Fields]) AND ("children"[All Fields] OR "child"[All Fields] OR "schoolchildren"[All Fields] OR "young"[All Fields] OR "youth"[All Fields] OR "adolescents"[All Fields] OR "young people"[All Fields] OR "teenagers"[All Fields])).

Table S2. Search strategy for Embase (140).

Database/platform	Date(year-month-day)
Embase	Start to End:<br>1883-01-01 to 2025-11-03

(children:ti,ab,kw OR child:ti,ab,kw OR schoolchildren:ti,ab,kw OR young:ti,ab,kw OR youth:ti,ab,kw OR adolescents:ti,ab,kw OR 'young people':ti,ab,kw OR teenagers:ti,ab,kw) AND ('free fat mass':ti,ab,kw OR 'fat mass':ti,ab,kw OR 'body mass index':ti,ab,kw OR 'lean body mass':ti,ab,kw OR 'body fat percentage':ti,ab,kw OR 'visceral fat':ti,ab,kw OR 'body weight':ti,ab,kw OR overweight:ti,ab,kw OR obesity:ti,ab,kw) AND (boxing:ti,ab,kw OR judo:ti,ab,kw OR karate:ti,ab,kw OR taekwondo:ti,ab,kw OR fencing:ti,ab,kw OR wrestling:ti,ab,kw OR 'olympic combat sports':ti,ab,kw) AND [01-01-1883]/sd NOT [04-11-2025]/sd.

Table S3. Search strategy for ProQuest(10）Database：APA PsycArticles ；APA Psyclnfo.

Database/platform	Date(year-month-day)
ProQuest	Start to End:<br>1900-01-01 to 2025-11-03

((subject(boxing) OR subject(fencing) OR subject(judo) OR subject(karate) OR subject(taekwondo) OR subject(wrestling) OR subject(Olympic combat sports)) AND (subject(free fat mass) OR subject(fat mass) OR subject(body mass index) OR subject(lean body mass) OR subject(body fat percentage) OR subject(visceral fat) OR subject(body weight) OR subject(overweight) OR subject(obesity)) AND (subject(children) OR subject(child) OR subject(schoolchildren) OR subject(young) OR subject(youth) OR subject(adolescents) OR subject(young people) OR subject(teenagers))).

Table S4. Search strategy for Web of Science (677).

Database/platform	Date(year-month-day)
Web of Science	Start to End:<br>1985-01-01 to 2025-11-03

[Topic](“boxing” OR “fencing” OR “judo” OR “karate” OR “taekwondo” OR “wrestling” OR “Olympic combat sports”) AND [Topic] (“free fat mass” OR “fat mass” OR “body mass index” OR “lean body mass” OR “body fat percentage” OR “visceral fat” OR “body weight” OR “overweight” OR “obesity”) AND [Topic](“children” OR “child” OR “schoolchildren” OR “young” OR “youth” OR “adolescents” OR “young people” OR “teenagers”).

Table S5. Search strategy for EBSCOhost (70)Database: CINAHL Ultimate; MEDLINE Complete；ERIC；SPORTDiscus with Full Text.

Database/platform	Date(year-month-day)
EBSCOhost	Start to End:<br>1900-01-01 to 2025-11

[Subject Terms](“boxing” OR “fencing” OR “judo” OR “karate” OR “taekwondo” OR “wrestling” OR “Olympic combat sports”) AND [Subject Terms] (“free fat mass” OR “fat mass” OR “body mass index” OR “lean body mass” OR “body fat percentage” OR “visceral fat” OR “body weight” OR “overweight” OR “obesity”) AND [Subject Terms](“children” OR “child” OR “schoolchildren” OR “young” OR “youth” OR “adolescents” OR “young people” OR “teenagers”).
